# Development and validation of a deep learning model for breast lesion segmentation and characterization in multiparametric MRI

**DOI:** 10.3389/fonc.2022.946580

**Published:** 2022-08-11

**Authors:** Jingjin Zhu, Jiahui Geng, Wei Shan, Boya Zhang, Huaqing Shen, Xiaohan Dong, Mei Liu, Xiru Li, Liuquan Cheng

**Affiliations:** ^1^ School of Medicine, Nankai University, Tianjin, China; ^2^ Department of General Surgery, Chinese People’s Liberation Army General Hospital, Beijing, China; ^3^ Department of Neurology, Beijing Tiantan Hospital, Beijing, China; ^4^ Department of Neurology, Beijing Tiantan Hospital, Capital Medical University, Beijing, China; ^5^ Department of Radiology, Chinese People’s Liberation Army General Hospital, Beijing, China; ^6^ Department of Pathology, Chinese People’s Liberation Army General Hospital, Beijing, China

**Keywords:** breast, magnetic resonance imaging, convolutional neural networks, deep learning, artificial intelligence

## Abstract

**Importance:**

The utilization of artificial intelligence for the differentiation of benign and malignant breast lesions in multiparametric MRI (mpMRI) assists radiologists to improve diagnostic performance.

**Objectives:**

To develop an automated deep learning model for breast lesion segmentation and characterization and to evaluate the characterization performance of AI models and radiologists.

**Materials and methods:**

For lesion segmentation, 2,823 patients were used for the training, validation, and testing of the VNet-based segmentation models, and the average Dice similarity coefficient (DSC) between the manual segmentation by radiologists and the mask generated by VNet was calculated. For lesion characterization, 3,303 female patients with 3,607 pathologically confirmed lesions (2,213 malignant and 1,394 benign lesions) were used for the three ResNet-based characterization models (two single-input and one multi-input models). Histopathology was used as the diagnostic criterion standard to assess the characterization performance of the AI models and the BI-RADS categorized by the radiologists, in terms of sensitivity, specificity, accuracy, and the area under the receiver operating characteristic curve (AUC). An additional 123 patients with 136 lesions (81 malignant and 55 benign lesions) from another institution were available for external testing.

**Results:**

Of the 5,811 patients included in the study, the mean age was 46.14 (range 11–89) years. In the segmentation task, a DSC of 0.860 was obtained between the VNet-generated mask and manual segmentation by radiologists. In the characterization task, the AUCs of the multi-input and the other two single-input models were 0.927, 0.821, and 0.795, respectively. Compared to the single-input DWI or DCE model, the multi-input DCE and DWI model obtained a significant increase in sensitivity, specificity, and accuracy (0.831 vs. 0.772/0.776, 0.874 vs. 0.630/0.709, 0.846 vs. 0.721/0.752). Furthermore, the specificity of the multi-input model was higher than that of the radiologists, whether using BI-RADS category 3 or 4 as a cutoff point (0.874 vs. 0.404/0.841), and the accuracy was intermediate between the two assessment methods (0.846 vs. 0.773/0.882). For the external testing, the performance of the three models remained robust with AUCs of 0.812, 0.831, and 0.885, respectively.

**Conclusions:**

Combining DCE with DWI was superior to applying a single sequence for breast lesion characterization. The deep learning computer-aided diagnosis (CADx) model we developed significantly improved specificity and achieved comparable accuracy to the radiologists with promise for clinical application to provide preliminary diagnoses.

## 1 Introduction

Multiparametric magnetic resonance imaging (mpMRI) consisting of functional imaging techniques such as dynamic contrast-enhanced MRI (DCE-MRI) and diffusion-weighted imaging (DWI) is widely used for the screening, diagnosis, and preoperative evaluation of breast diseases (1–3). Several studies have demonstrated that mpMRI can provide complementary morphology and function data for the discrimination of benign and malignant breast tumors, significantly improving the diagnostic accuracy in breast cancer and reducing unnecessary breast biopsies of benign lesions (4–7).

However, mpMRI produced massive amounts of image data with a high spatial and temporal resolution, and radiologists face challenges in the correct interpretation (2, 8). Although the US Breast Imaging-Reporting and Data System (BI-RADS) lexicon provides a structured common language for interpretation and reporting, the defined rules for converting specific imaging features into a diagnostic category are not available (9). Most researchers employed multivariate logistic regression analysis to determine imaging features that jointly are associated with malignancy (6, 7, 10, 11).

With the advances in computer technology, extracting large amounts of data from medical images using automatic algorithms becomes more feasible (12, 13). In particular, deep learning (DL) algorithms based on convolutional neural networks (CNNs) for image analysis have achieved prominence for lesion characterization in breast MRI (14–16). The current methods always use manual annotation to identify and delineate lesions although it is a tedious and time-consuming process and leads to deviations in the presence of background parenchymal enhancement (BPE) (17–19).

Therefore, the purpose of our study was to develop a fully automated DL computer-aided diagnosis (CADx) model for breast lesion accurate segmentation and characterization. Firstly, using CNNs to segment the breast lesions from the DCE and DWI. Then, based on the segmentation results, we designed two single-input models using DCE and DWI as input sequences, respectively, and a multi-input model combining the two sequences to differentiate benign and malignant lesions and compare the performance of the AI models and the BI-RADS categorized by the radiologists. Finally, we collected data from another medical center to test the robustness of the proposed models.

## 2 Related works

DL is a type of machine learning with hundreds of deep layers of neural networks. Each layer learns to detect features of increasing complexity from the images and then combines lower-level features to form more abstract higher-level representational attributions or features to discover distributional features of the data (20). In contrast to traditional machine learning (ML) methods, DL can learn directly by navigating the data space without feature engineering and achieve an end-to-end result output (21). Consequently, more medical image research studies are focusing on DL methods, and such methods have outperformed most traditional ML methods. The study of DL in breast MRI mainly focuses on lesion detection and segmentation, characterization and classification, and radiogenomics.

### 2.1 Lesion identification and segmentation

Segmentation is a highly relevant task in medical image analysis. One of the earliest works was made by Cirean et al. (22). The network predicts the category label of each pixel by providing the local region (patch) around it as input. Due to a large number of overlapping patches, the network must run separately for each patch, leading to extremely slow operation. Furthermore, there is a trade-off between localization accuracy and the use of context. Subsequently, Ronneberger et al. (23) modified and extended the fully convolutional network architecture. The new network adopted a U-shaped architecture without any fully connected layers and used only the effective part of each convolution, i.e., the segmentation map contains only the pixels available in the full context of the input image. In addition, the authors preserved extensive feature map channels so that more information can flow into the final recovered segmented images, which allows the network suitable for fewer training images and produces more accurate segmentation. Up to now, UNet is always the basic network architecture for image segmentation tasks. Considering that diagnostic and interventional images in medicine are often volumetric, researchers further explored segmentation algorithms applicable to 3D images. Çiçek et al. (24) and Milletari et al. (25) designed 3DUNet and VNet, respectively, by replacing all 2D operations with corresponding 3D operations in UNet or adding a new loss layer specifically designed for the segmentation task based on Dice similarity coefficient (DSC) to UNet.

DCE-MRI includes the temporal acquisition of 3D volumes before and after intravenous injection of paramagnetic contrast agent, hence having four-dimensional data, which is essential for breast lesion analysis. The traditional UNet fails to capture temporal information. To overcome this drawback, Chen et al. (26) constructed a spatiotemporal network by modifying the standard U-shaped network and adding a novel convolutional long short-term memory (ConvLSTM) structure for extracting spatiotemporal information while preserving high spatial resolution. In addition, the 3TP UNet, which utilizes a three-time point approach to improve lesion segmentation performance, was proposed and optimized. Results showed that the network outperforms classical and some new deep learning methods with an average DSC exceeding 60%, laying the foundation for a protocol-independent approach (27, 28).

### 2.2 Lesion characterization and classification

Breast lesion diagnosis belongs to a classification task. At this clinical step, multiple breast modalities are often used and integration of the findings is required (29). Conventional MRI examinations perform direct diagnosis mainly on the basis of morphological features of breast lesions, while DCE-MRI is based on rapid scanning imaging sequences and uses pharmacokinetic models to determine the intra- and extravascular temporal intensity profiles of contrast agents and to analyze changes in perfusion, microcirculation, and capillary permeability at the lesion site. Thus, current clinical image analysis systems are mainly concerned with displaying enhanced regions and their voxel-by-voxel kinetic profiles and corresponding threshold levels.

Herent et al. (30) created a lesion feature model using a 50-layer residual neural network based on a single two-dimensional T1-weighted fat-suppressed MRI obtained after intravenous injection of gadolinium chelate selected by the radiologist, reaching a weighted mean area under the receiver operating characteristic (ROC) curve (AUC) of 0.816 on the independent challenge test set. Zhou et al. (31) and Antropova et al. (32) compared the diagnostic performance for benign and malignant categorization of lesions in DCE-MRI by radiomics analysis and CNN models based on ResNet50 and VGG19, respectively. Significant improvements in predictive performance in assessing the malignancy of lesions were observed in DL compared to handcrafted features. To optimize the structure of the CNN model, Hizukuri et al. (33) initially identified a baseline model from AlexNet, ZFNet, VGG16, and GoogLeNet in terms of the AUC. Afterward, the hyperparameters in the baseline model, such as the number of convolutional layers, the number of filters, and the size of the filters, were optimized using the Bayesian optimization with the Gaussian process. The model achieved high classification performance with accuracy, sensitivity, specificity, positive predictive value, and negative predictive value of 92.9%, 93.3%, 92.3%, 93.3%, and 92.3%, respectively. However, it should be noted that the model was designed only for the differential diagnosis of breast masses.

In addition, it has been investigated to incorporate dynamic and volumetric components of DCE-MRI into breast lesion classification using maximum intensity projection (MIP) images, and the results demonstrated that combining volumetric and dynamic DCE-MRI components can significantly improve CNN-based lesion classification (34, 35). For more comprehensive use of the information contained in MRI, some studies have explored the addition of DWI and T2-weighted volumes along with DCE-MRI to improve the specificity of clinical breast MRI protocols further (2, 36–39). However, the relevant studies employing DL methods are fewer.

### 2.3 Radiogenomics

Several state-of-the-art models, which exploit MRI images, have been developed for other purposes, such as differentiating the molecular subtypes of breast cancer and predicting breast cancer recurrence and pathologic complete response in patients receiving adjuvant therapy. Zhu et al. (40) investigated three different deep learning methods to conduct radiogenomic analysis of breast cancer: training from scratch, transfer learning, and off-the-shelf deep features. The best AUC performance was achieved by the off-the-shelf deep features approach with 0.65 (95% CI, [0.57, 0.71]). Another study applied a traditional CNN and a recurrent network using ConvLSTM. When the developed models were tested on an independent dataset, the accuracy was 0.4–0.5. Then, by re-tuning the models through transfer learning, the overall classification accuracy was improved by greater than 30% (41).

Assessing the efficacy of treatment is also an important clinical application of radiogenomics. It is expected to reveal the prognostic connection between imaging and patients by fusing imaging and genetic and pathological features. So far, there have been several attempts to develop CNN-based approaches with the objective of predicting pathological complete response (pCR) in breast patients using pretreatment MRI examinations, and the models all achieved an average AUC of more than 70% (42–45).

## 3 Materials and methods

This cross-sectional, retrospective, multicenter study was approved by the Ethics Committee of the Chinese People’s Liberation Army (PLA) General Hospital (No. S2019-093-01), and the requirement for individual consent was waived due to the retrospective nature of the analysis.

### 3.1 Study dataset

**Figure 1:**
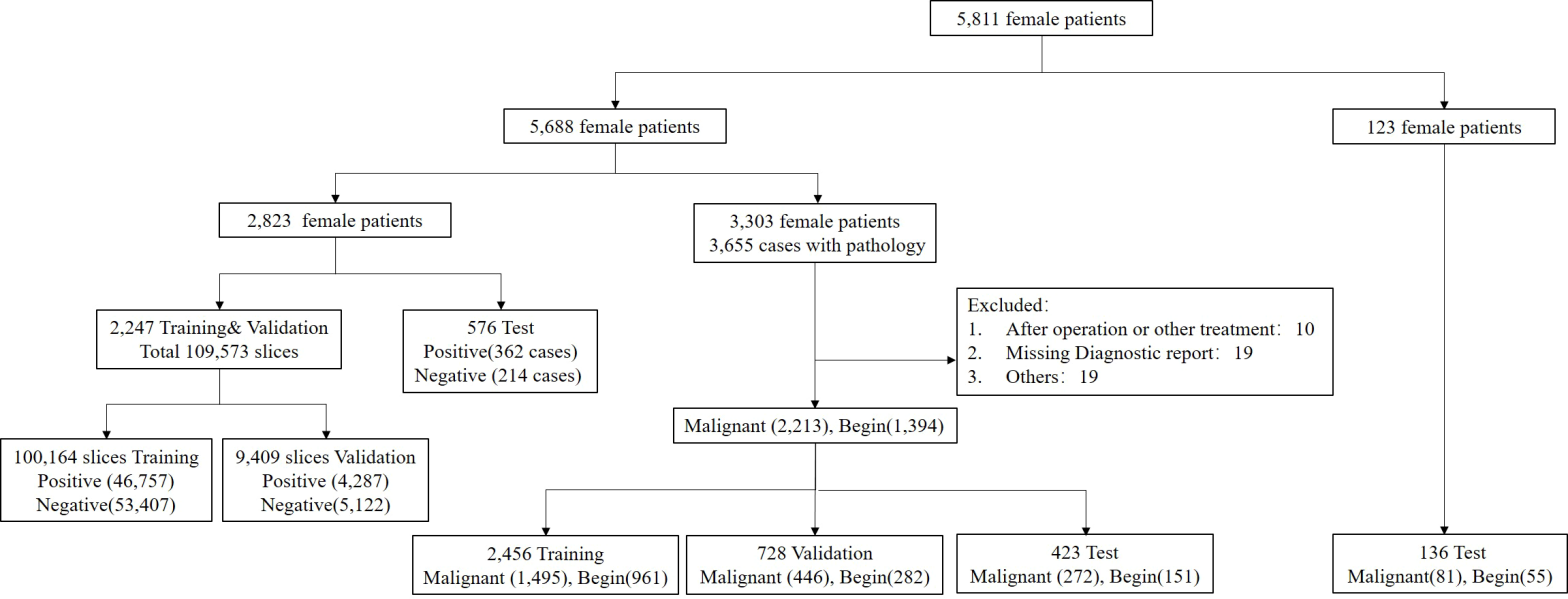
Flowchart of the final analysis cohort. A total of 5,811 patients were included in this study. A total of 5,688 patients within our institution were used for the segmentation and characterization model training, validation, and testing, and 123 patients from another institution were used for external testing of the proposed characterization model.

A total of 5,688 female patients undergoing breast mpMRI in the 1st Medical Center of PLA General Hospital from January 2011 to May 2020 were retrospectively enrolled. There were 2,823 patients who were randomly selected for training, validation, and testing of the segmentation model, and 3,303 patients with the original BI-RADS classification and pathological validation were used to train, validate, and test the characterization model (**Figure 1**). For the characterization task, the unit for the calculation and statistics was named “breast,” and each patient had two breasts. A breast is defined as benign if only benign lesions were present in it, malignant if only malignant lesions were present in it, or malignant if both benign and malignant lesions were co-existing in it. After excluding 48 lesions with an exhibited evident mismatch of location in the MRI report and surgery or biopsy or with a history of surgical intervention or other treatment prior to MRI examination, the study included 3,303 female patients with 3,607 pathologically confirmed lesions (2,213 malignant and 1,349 benign). Another 123 female patients with 81 pathologically confirmed malignant lesions and 55 benign lesions were collected from the 6th Medical Center from July 2020 to September 2021. The MRI protocol was attached to the **Supplement**.

### 3.2 Deep learning system

**Figure 2:**
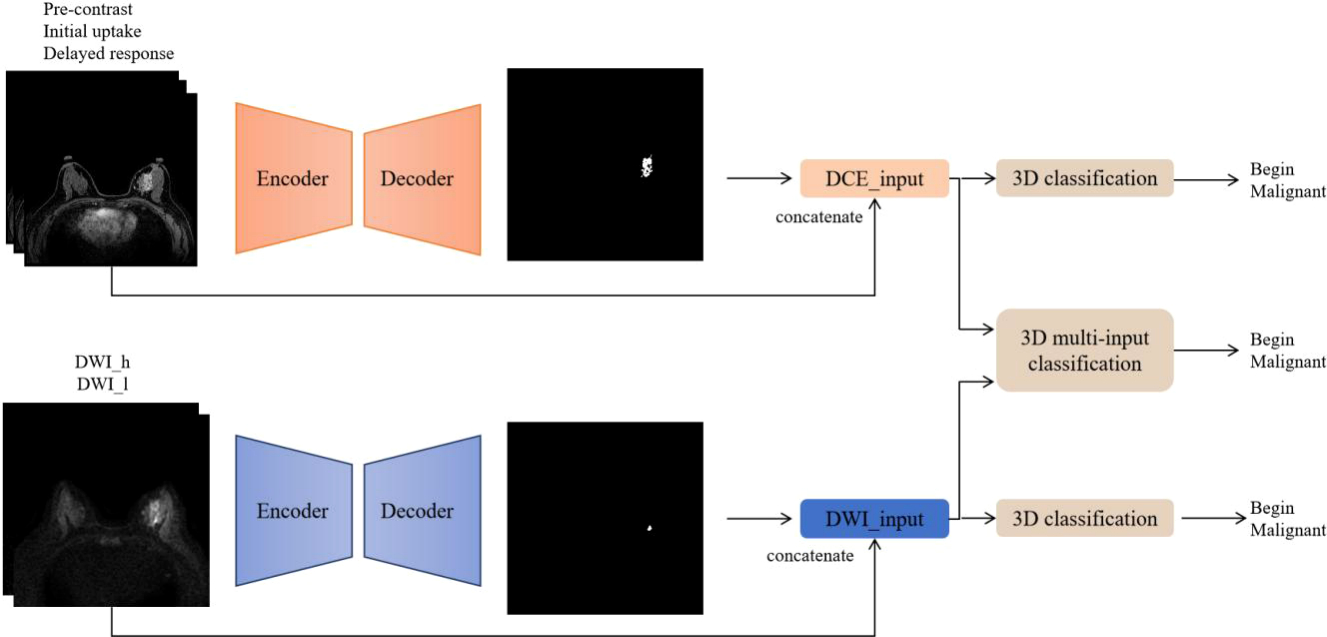
Flowchart of the deep learning algorithm. DCE and DWI were respectively input into the AI model to obtain lesion segmentation results and preliminary diagnosis. Then, the images from the two sequences were integrated and input into the 3D multi-input model to obtain the ultimate diagnosis.

The code implementations were in-house developments based on Python 3.6.8 (https://www.python.org), and the software modules numpy, scipy, pandas, and sklearn were utilized. **Figure 2** summarizes the workflow of the DL algorithm, consisting of the following two steps: 1) imaging segmentation of breast lesions and 2) lesion characterization of benign and malignant.

#### 3.2.1 Imaging segmentation of breast lesions

A 2D VNet framework was implemented for DCE imaging of lesion segmentation (23, 25). On DCE sequence, three phases, the precontrast, initial uptake, and 8–10 min delay, were selected as the input of the model, with the size of 512 * 512 * 3. Since the number of slices of DWI is less than that of DCE due to the influence of thickness, the segmentation model of DWI adopted the Attention-UNet (46). The high *b*-value and low *b*-value images of DWI were input into the network as different channels, with the size of 256 * 256 * 2. Both VNet and Attention-UNet used the same segmentation loss function. The loss function is a combination of binary cross-entropy (BCE) and DSC on each of the above four semantic levels, which is described as


$$Lossfunction=\frac{1}{2}BCELoss+\frac{1}{2}DiceLoss$$

$$BCELoss=-\frac{1}{M}\sum_{i=1}^{M}\left(Y_{i}\cdot log\left(\hat{Y_{i}}\right)+\left(1-Y_{i}\right)\cdot log\left(1-\hat{Y_{i}}\right)\right)$$

$$DiceLoss=1-\frac{2\sum_{i}^{N}p_{i}g_{i}}{\sum_{i}^{N}p_{i}^{2}+\sum_{i}^{N}g_{i}^{2}}$$

where *Y_i_* is the ground truth and ${\hat{Y}}_{i}$ is the predicted probability for all the *M* pixels and the sums run over the *N* voxels of the predicted binary segmentation volume *p_i_* ∈*P* and the ground truth binary volume *g_i_* ∈*G*.

In the training set, 80 epochs in total with Adam optimization and a momentum of 0.9 were used. Due to the limitation of GPU memory, the batch size parameter was set to 16. The initial learning rate was 0.0001 and multiplied by 0.1 every 6 epochs. In the inference subset, a breast MRI volume can be segmented by processing it in a feedforward manner through the network. The output of the last convolutional layer, after sigmoid, consists of a probability map for background and foreground. The voxels having a higher probability (>0.5) that belong to the foreground than to the background are considered part of the anatomy.

#### 3.2.2 Lesion characterization of benign and malignant

**Figure 3:**
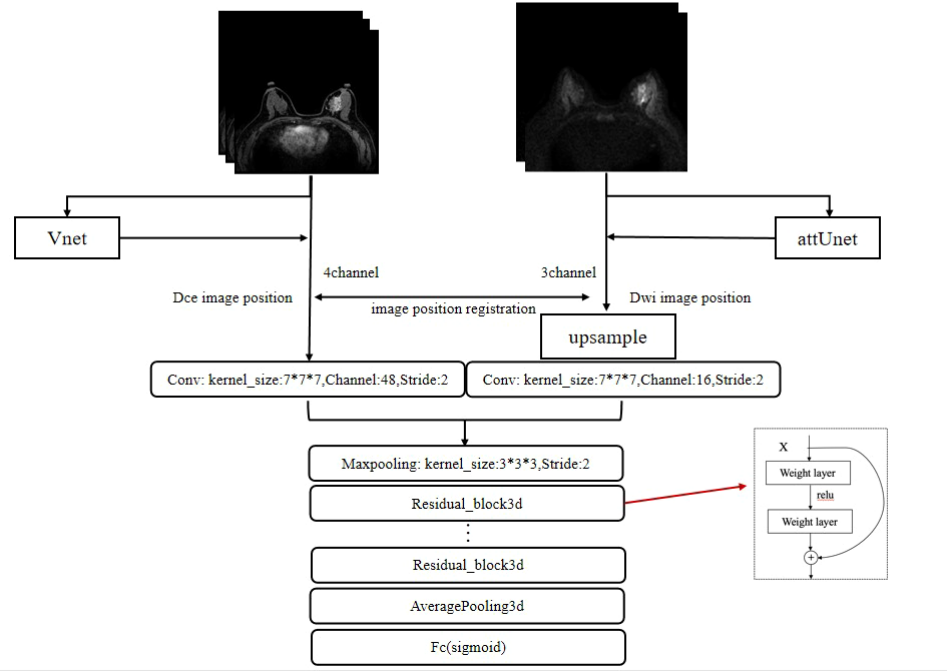
The framework for the segmentation of the breast images and the characterization of lesions. The whole framework is divided into two parts. The upper part represents the image segmentation process. By inputting images into the segmentation networks, such as VNet and attUNet, the mask of the breast region is obtained. By applying the mask to the input image, the breast lesion can be segmented. The following part represents the target characterization. The DCE and DWI sequences of the unilateral breast are input to the multi-input model to obtain the probability of malignancy.

A 3D version of ResNet was implemented for lesion characterization (47). The final workflow schematic is shown in **Figure 3**. Considering that DCE contains more lesion information than DWI, 48 filters were set for DCE and 16 filters were set for DWI and then concatenated to form 64 filters. The network consists of 4 Residual blocks, and the basic block was repeated 3, 4, 6, and 3 times within the Residual blocks, respectively. The basic block consists of 3 * 3 * 3 convolution, Relu, Batch Normalization layer, and a shortcut connection. A single dense layer with a sigmoid activation function was used to generate the final likelihood values. To initialize the weight parameters of the network, He initialization was used (48). Since the models are easy to get overfitted and the scanning mode can be widely ranged among radiologists, to improve the generalization performance and robustness of our models, we changed the image orientation by implementing a combination of randomly flip and rotate on our input images as data augmentation. Flip is to turn over the images horizontally and vertically along the given axis, and rotation is to turn about the images around 90° along the specified axis. To optimize the weights, we used stochastic gradients with Adam momentum of 0.9. During training, we used a batch size of 12 and an initial learning rate of 0.001, divided by 10 after the 5th epoch.

To compare the effects of different sequences on the characterization of breast lesions, DCE, DWI, DCE and DWI, and their corresponding segmentation results were used as inputs to the model, respectively, and thus, two single-input models and one multi-input model were obtained. The images were organized into two categories and tested using the three learned models to obtain the benign or malignant labels. In addition, Grad-CAM, a visualization method, was used to create a heat map that highlights the essential parts of the input image that are considered important in each block for character distinction (49).

### 3.3 Statistical evaluation

For lesion segmentation, the manual segmentation results by the radiologist in DCE were defined as the ground truth, and the DSC was used to perform a quantitative comparison between the model and the ground truth (50).

For lesion characterization, histopathology was used as the diagnostic criterion standard to analyze the sensitivity, specificity, positive predictive value (PPV), negative predictive value (NPV), diagnostic accuracy, and ROC curves with AUCs of the models and radiologists. In assessing the performance of radiologists, the BI-RADS category was dichotomized. BI-RADS 2 and 3 were considered as benign and BI-RADS 4 and 5 were considered as malignant. Moreover, considering that BI-RADS 4 had a wide range of probabilities of malignancy, extending from greater than 2% to less than 95% (51), we decided to include such type of lesions as benign for reassessment. All statistical analyses were performed with Python 3.6.8 statistic modules. Standard deviations and confidence intervals were calculated by using bootstrap analysis with 100,000-fold resampling as in Litjens et al. (52). For clinical evaluation, the McNemar test was used to compare the categorical characterization results of the three models and radiologists with *p*-values <0.05 considered statistically significant.

## 4 Results

### 4.1 Study population and lesion characteristics

The internal dataset analysis cohort consisted of bilateral breast mpMRI datasets from 3,303 patients [mean age, 45.4 ± 11.3 years (standard deviation); range, 11–89 years] and 304 patients have bilateral breast lesions. In total, there were 3,607 enhancing lesions identified and segmented: 1,394 (38.6%) were benign and 2,213 (54.6%) were malignant. Detailed pathological information of the lesions included in the dataset is summarized in **Table 1**.

**Table 1:** The characteristics and pathological types of patients included in the study.

Parameter	Internal dataset				External dataset
	Total	Training	Validation	Test	
**Age**					
Mean (range)	46.09 (11~89)	45.92 (11~87)	46.16 (13~85)	46.84 (20~89)	48.45 (27~80)
**Number**					
Women	3,303	2,339	724	469	123
**Pathology**					
*Benign*	*1,394*	*961*	*282*	*151*	*56*
Adenosis	584	402	122	60	5
Fibroadenoma	370	254	76	40	30
Mastitis	165	103	41	21	4
Intraductal papilloma	224	163	36	25	14
Phylloid tumor	49	38	6	5	3
Hamartoma	2	1	1	0	0
*Malignant*	*2,213*	*1,495*	*446*	*272*	*82*
Medullary carcinoma	10	8	2	0	2
Mucinous carcinoma	37	23	11	3	2
Carcinoma *in situ*	212	150	35	27	8
Invasive carcinoma	1,954	1,314	398	242	70

### 4.2 Imaging segmentation

**Figure 4:**
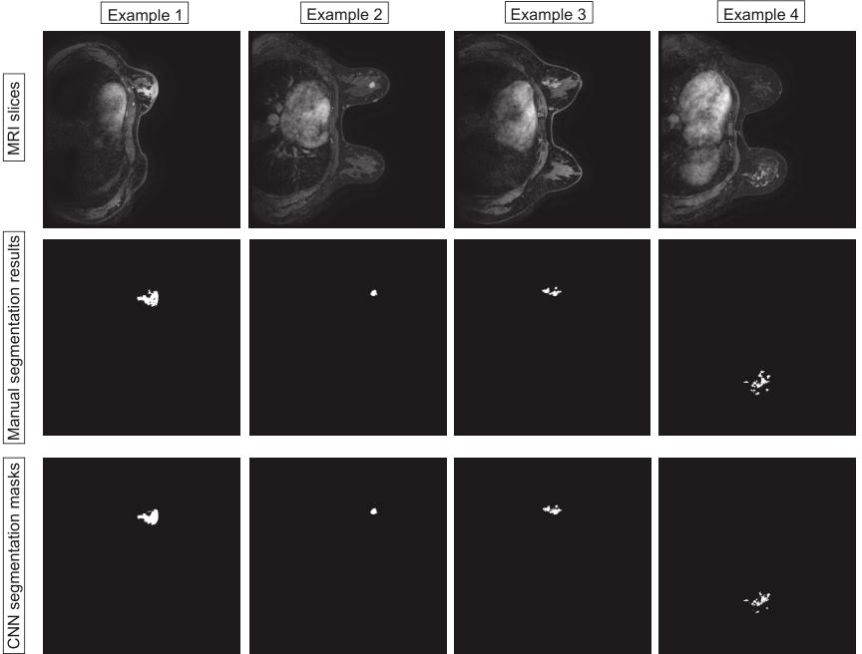
Examples of the 2D breast lesion segmentation results. Examples 1 and 2: the fibroglandular structure was dense and the lesions showed mass-like enhancement with an irregular lobulated pattern and hairy margins. Examples 3 and 4: the lesions showed non-mass-like enhancement with a distribution characterized by lobular segments and internal enhancement characterized by inhomogeneous string–ring-like and patch-like features, respectively.

In this section, we compare the segmentation method based on VNet with UNet and Attention-UNet. The VNet architecture achieved the highest DSC of 0.860 with precision and recall of 0.867 and 0.853, respectively. Furthermore, the statistical results of all comparison methods are summarized in **Table 2**. **Figure 4** shows example images of the 2D breast lesion segmentation results, the masks produced by the VNet were quite similar to the region segmented manually, and significant structural details of the lesions were further preserved.

**Table 2:** The results of the segmentation models.

Method	DSC	Precision	Recall
VNet	0.860	0.867	0.853
Attention-UNet	0.829	0.815	0.843
UNet	0.802	0.800	0.804

### 4.3 Model visualization

**Figure 5:**
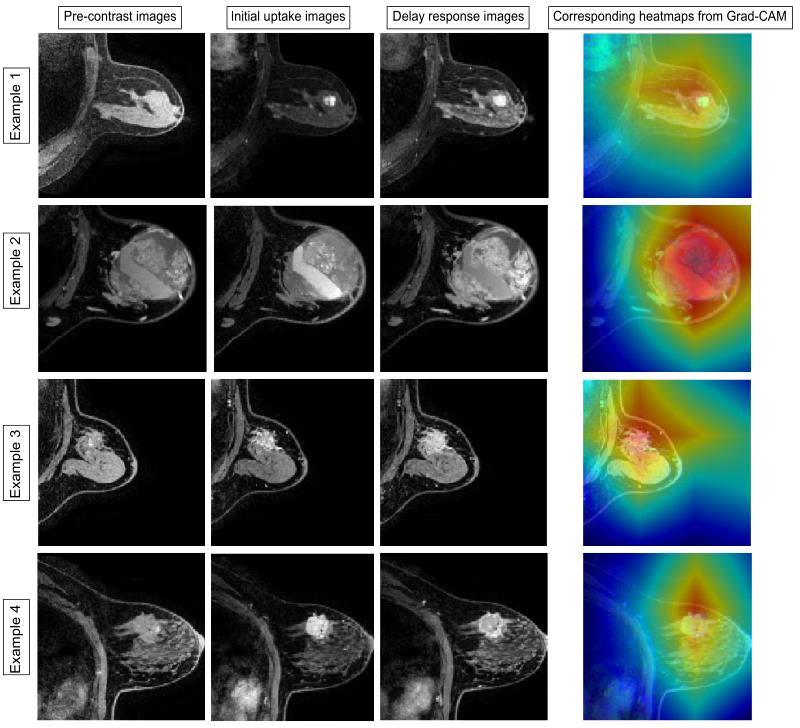
Examples of the attended regions through the Grad-CAM. The pathological types of the four cases were adenosis, benign lobular tumor, ductal carcinoma *in situ*, and invasive carcinoma. With the visual model display, the brighter area that contains lesion extent can be observed, demonstrating that the classification model can focus on the lesion area effectively.

Two malignant tumors and one benign tumor correctly diagnosed by the model were selected to visualize the regions of interest. From **Figure 5**, it can be observed that the heat maps generated from the Grad-CAM are able to locate the lesion areas with higher activations than normal areas, with deeper color indicating higher activation, which presented an intuitive interpretation of what the models have learned from the training data and proved the networks can be automatically driven to focus on the lesions.

### 4.4 Lesion characterization

The sensitivities, specificities, PPVs, NPVs, diagnostic accuracies, AUCs, and the corresponding 95% CI for the three models and radiologists are summarized in **Table 3**.

**Table 3:** The diagnostic performances of the characterization models and radiologists.

Method (internal test results/external test results)	Specificity (95% CI)	Sensitivity (95% CI)	PPV (95% CI)	NPV (95% CI)	Accuracy (95% CI)	AUC (95% CI)
DWI	0.629 (0.528, 0.728)	0.772 (0.704, 0.837)	0.790 (0.724, 0.852)	0.610 (0.505, 0.702)	0.721 (0.664, 0.776)	0.795 (0.738–0.847)
	0.768 (0.681, 0.846)	0.805 (0.740, 0.866)	0.836 (0.772, 0.893)	0.729 (0.643, 0.811)	0.790 (0.736, 0.840)	0.831 (0.779–0.880)
DCE	0.709 (0.612, 0.802)	0.776 (0.709, 0.840)	0.827 (0.765, 0.886)	0.630 (0.541, 0.731)	0.752 (0.696, 0.804)	0.821 (0.766–0.873)
	0.839 (0.763, 0.907)	0.732 (0.658, 0.803)	0.869 (0.807, 0.926)	0.681 (0.598, 0.763)	0.775 (0.724, 0.824)	0.812 (0.756–0.865)
DCE and DWI	0.874 (0.800, 0.939)	0.831 (0.772, 0.887)	0.926 (0.875, 0.965)	0.741 (0.654, 0.821)	**0.846 (0.800, 0.888)**	**0.927 (0.893–0.956)**
	0.875 (0.806, 0.936)	0.731 (0.660, 0.801)	0.896 (0.838, 0.947)	0.690 (0.610, 0.796)	**0.790 (0.740, 0.840)**	**0.885 (0.842–0.922)**
BI-RADS by radiologists 2, 3 and 4, 5	0.404 (0.302, 0.510)	0.978 (0.952, 1.00)	0.747 (0.687, 0.805)	0.911 (0.813, 1.00)	0.773 (0.720, 0.824)	0.691 (0.638–0.744)
	0.571 (0.473, 0.667)	0.963 (0.929, 0.993)	0.767 (0.705, 0.826)	0.911 (0.813, 1.00)	0.804 (0.752, 0.805)	0.767 (0.717–0.813)
BI-RADS by radiologists 2–4 and 5	0.841 (0.760, 0.914)	0.904 (0.856, 0.947)	0.911 (0.864, 0.952)	0.830 (0.750, 0.904)	**0.882 (0.840, 0.920)**	0.873 (0.827–0.916)
	0.804 (0.723, 0.879)	0.878 (0.823, 0.928)	0.867 (0.740, 0.890)	0.818 (0.750, 0.904)	**0.848 (0.800, 0.892)**	0.841 (0.792–0.885)

PPV, positive predictive value; NPV, negative predictive value; 95% CI, 95% confidence intervals; AUC, the area under the curve; DWI, diffusion-weighted imaging; DCE, dynamic contrast enhancement; BI-RADS, Breast Imaging-Reporting and Data System. Bold indicates better results.

For the internal test, in the three models of single-input DWI or DCE and multi-input mpMRI, the overall accuracies were 0.721, 0.752, and 0.846, respectively. Compared with the other two single-input models, the multi-input model obtained the highest sensitivity of 0.831, specificity of 0.874, and accuracy of 0.846. In addition, by increasing the input sequence, the PPV of the model increased from 0.827 to 0.926 and the NPV increased from 0.630 to 0.741. For the external test, the single-input DWI and multi-input models were almost identical in terms of accuracy with 0.790, outperforming the single-input DCE of 0.775. The multi-input model achieved the highest specificity of 0.875, but the highest sensitivity was obtained by the single-input DWI model of 0.805.

The performance of the radiologists was also evaluated. When both categories 4 and 5 were classified as malignant, the sensitivity, specificity, and accuracy were 0.978, 0.404, and 0.773, respectively. When category 5 was classified as malignant, the sensitivity, specificity, and accuracy were 0.904, 0.841, and 0.882, respectively. Regardless of whether using BI-RADS category 3 or 4 as the cutoff point, radiologists had higher sensitivity with statistical significance than either of the models.

Further comparisons were performed to assess whether there are differences between the model and the radiologist’s characterization methods in clinical terms (**Supplementary Table 1**). There was no statistically significant difference between single-input DCE or DWI and multi-input mpMRI in the characterization results (*p* = 0.057, *p* = 0.260). Multi-input mpMRI and two classifications of diagnostic results by radiologists both have different results with higher detection rates by radiologists (*p* < 0.001, *p* = 0.003).

**Figure 6:**
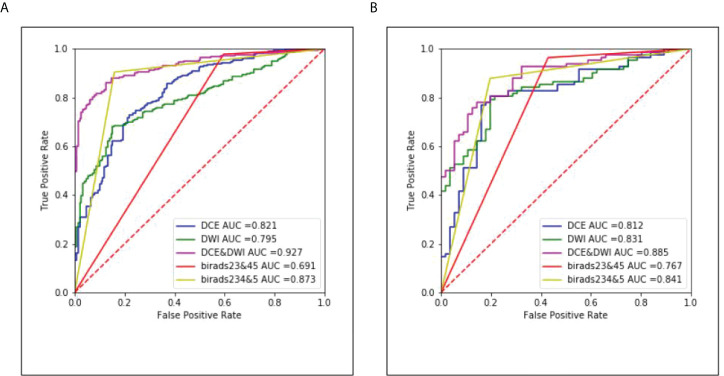
The corresponding ROC curves of the three different models and radiologists. **(A)** Results of the internal test set and **(B)** results of the external test set.

The ROC curves of the classification task in the three different models and radiologists are given in **Figure 6**. The multi-input model obtained the highest AUC values in both internal and external tests: 0.927 (95% CI, 0.893–0.956) and 0.885 (95% CI, 0.842–0.922), respectively.

## 5 Discussion

In this study, we developed a CADx model, combining a VNet-based segmentation model and a ResNet-based classification model for implementing benign and malignant characterization of breast lesions in mpMRI. The multi-input model achieved the best classification performance with an accuracy of 0.846 and an AUC of 0.927, demonstrating the superiority of multiple sequences over a single sequence. Moreover, in comparison with the characterization results of the radiologists, it obtained a generally comparable accuracy while significantly improving the specificity.

Previous research studies have reported the usefulness of DL for diagnostic imaging of breast lesions with MRI (17, 18, 31, 53–55). Truhn et al. (18) constructed a deep residual neural network (ResNet18) for the characterization of enhancing lesions in MRI and achieved superior performance compared to radiomic analyses. In their study, the radiologist must first identify the lesion to make use of the system, yet this process may lead to detection errors. Zhou et al. (54) used the entire segmented breast as input to predict the presence of lesions inside. The main innovation was to localize the lesion, but their model could only detect lesions with a high probability of malignancy. In our study, two CNN models were firstly used to identify and segment lesions prior to diagnosis, reducing the time radiologists take to review images to locate lesions, especially for non-mass-like enhancements. We also compared the sample size and DSC with other relevant research on breast lesion segmentation (56–60). Maicas et al. (59) proposed a segmentation method that combined global inference in the continuous space with deep learning for the problem of breast mass segmentation from DCE-MRI and obtained a DSC of 0.77. The study of Zhang et al. (56) also included only mass-type lesions. The difference was that they customized a 3D model in addition to a 2D model to take advantage of the potential spatial information of MRI volume. Compared to the 2D model, the 3D model achieved slightly better performance in terms of the DSC on the same dataset. In order to integrate the advantages of 2D and 3D networks, Wang et al. (58) proposed a mixed 2D and 3D convolutional network with a multiscale context (M2D3D-MC) for lesion segmentation with 90 studies and obtained a DSC of 0.77. This method focused on 2D as well as 3D information, but due to the limitation of data, the result of the DSC was not very good.

Since our AI diagnosis system used a tandem of the segmentation model and the classification model, we used 2D slices from three temporal phases of DCE images and DWI images with different *b*-values for segmentation and input the results into the classification model, which not only had a much lighter computation and higher calculation speed but also effectively preserved 3D contextual information. Furthermore, our study included 2,247 cases with 100,164 slices. Despite differences in the training datasets and some heterogeneity of MRI parameters, our model achieved comparable DSC performance similar to the published methods and is well-accepted by radiologists.

Currently, DCE is most commonly used in AI diagnostic models (33, 35, 61, 62). The addition of other sequences, such as DWI, to obtain higher diagnostic specificity has also started to be explored (63–65). Dalmiş et al. (17) investigated a DL model with an mpMRI protocol combining DCE-MRI, T2, and DWI. Unlike us, they used the apparent diffusion coefficient (ADC) value obtained from DWI and applied random forest to integrate the results. In comparison, considering that lesion heterogeneity is insufficiently described by a single ADC threshold, our model used the DWI images to retrieve more detailed structural and functional features and trained only one model to input multisequences, accomplishing lesion characterization. Hu et al. (55) also employed a CNN to extract and pool low- to mid-level features and trained a support vector machine classifier on CNN features to distinguish benign and malignant lesions. Their method involved more manual intervention without implementing a complete end-to-end process. Our multi-input characterization model adopted the early fusion method to learn the shallow information of the two sequences of DCE and DWI and fused the two sequences to learn the deeper information in the later stage. Although this approach differs from previous methods, it proves to be more simple, accessible, and effective, showing comparable diagnostic performance to the radiologists.

It is worth noting that there were misdiagnosis cases, both by the AI model and radiologists. We examined the findings of mpMRI, the results of radiologists, and the pathological features of these cases.

For false-positive (FP) lesions, the main pathological types of lesions produced by the AI model are mastitis and intraductal papilloma. These benign lesions may occasionally show suspicious features on breast MRI, but generally do not require specific treatment with minimal risk of future cancer development (66). Of the 21 cases of mastitis, the model misdiagnosed 10 cases, while the radiologists included 17 cases as category 4 or 5, compared to a mild improvement. Of the 25 cases of intraductal papillomas, the model misdiagnosed 5 cases, 3 of which were associated with atypical ductal hyperplasia (ADH), a low-grade neoplastic intraductal hyperplasia with varying risks of progression (67). Therefore, it is reasonable to classify them as malignant and recommend biopsy or other treatments. Overall, the automatic classification yielded 19 false positives, 47 fewer than the radiologists, effectively improving diagnostic specificity.

For false-negative (FN) lesions, the pathological types of lesions produced by the AI model were invasive carcinoma, ductal carcinoma *in situ*, and mucinous carcinoma. Lesions of invasive carcinoma with a maximum diameter of less than 1 cm were more likely to be recognized as benign lesions. In some of the cases, radiologists could make a correct diagnosis, but the AI system overlooks the lesions. Several factors may explain the discrepancy. When two or more lesions present in the breast simultaneously, especially in the coexistence of benign and malignant pathologies, the model may give average results, leading to an underestimation of the malignancy. In addition, a few lesions are accompanied by swollen lymph nodes, but this factor was not considered in our study. Mammography has obvious advantages in detecting ductal carcinoma *in situ* with microcalcification (68–70). Radiologists can combine mammogram results and perform clinical breast examinations for suspicious lesions, hence a higher accuracy. Finally, for the missed diagnosis of mucinous carcinoma, our main consideration was the included data were so little that the model may not be comprehensive in learning the characteristics of such lesions.

To sum up, our results showed that the multi-input model is beneficial for BI-RADS category 4 lesions by proving additional specificity. Therefore, we suggest that the added value of the current multi-input model could be as an adjunct decision-supporting tool for lesions of lower clinical suspicion to make a confident diagnosis of benignity, thus obviating biopsy intervention. In addition, paying attention to the characteristics of FN lesions and comprehending the disadvantages of the CNN model based on the results of this study may help radiologists use this model effectively and improve their diagnostic performance possibly.

Furthermore, the technical variability of scanning imaging has also been studied through another institution and different MRI scanners. In this external test, we observed a decrease in performance in terms of AUC of 4.2% points. Through retrospective analysis of FN and FP results, the error modes are similar to those of the previous internal test set. Therefore, we concluded that the model was robust and the decrease in its effectiveness was mainly due to the selection bias of the data. In the external dataset, the DWI single-input model showed an increase in AUC from 0.71 to 0.76 compared with the DCE. We attributed it to the fact that the DWI images in the external dataset were obtained with the *b*-value set to 1,000 s/mm2, resulting in a reduced image signal-to-noise ratio and better sharpness compared to the partial DWI images in the internal dataset with a b-value of 800sec/mm^2^.

It is important to acknowledge that this study has some limitations. First, since data from only two medical centers were utilized, it is difficult to evince from the presented results how the developed models might perform with data acquired under differing protocols. Future studies should focus on collecting representative, large, and multi-institutional datasets to test the CADx model. Second, we cannot determine whether the diagnostic performance of radiologists will be significantly improved with the aid of the multi-input model. Another observer study is necessary, in which two reading conditions would be evaluated: reading without AI aid and reading with AI aid. Lastly, our model uses only two breast MR sequences. It is necessary to design a complete automated diagnostic system incorporating other MRI sequences and patients’ clinical information.

## 6 Conclusion

The findings of this diagnostic trial demonstrated that the use of mpMRI in combination with DL had significantly improved the diagnostic performance and achieved acceptable diagnostic accuracy at the clinic level. It can be expected that the proposed DL-based CADx model can help radiologists by providing preliminary diagnosis, enabling greater efficiencies in interpreting breast mpMRI images.

## Data availability statement

The raw data supporting the conclusions of this article will be made available by the authors, without undue reservation.

## Ethics statement

The studies involving human participants were reviewed and approved by the Ethics Committee of Chinese People’s Liberation Army General Hospital (No. S2019-093-01). Written informed consent from the participants’ legal guardian/next of kin was not required to participate in this study in accordance with the national legislation and the institutional requirements.

## Author contributions

JZ and JG: study design, experiments, and manuscript writing. JZ, JG, WS, and HS: experiments, analysis, and interpretation of data. BZ, XD, and ML: collection and interpretation of data. XL and LC: interpretation of data and manuscript revision. All authors contributed to the article and approved the submitted version.

## Conflict of interest

The authors declare that the research was conducted in the absence of any commercial or financial relationships that could be construed as a potential conflict of interest.

## Publisher’s note

All claims expressed in this article are solely those of the authors and do not necessarily represent those of their affiliated organizations, or those of the publisher, the editors and the reviewers. Any product that may be evaluated in this article, or claim that may be made by its manufacturer, is not guaranteed or endorsed by the publisher.
