# Supplementary material for: Development and validation of a deep learning model for breast lesion segmentation and characterization in multiparametric MRI
Source: Front Oncol. 2022 Aug 11;12:946580. doi: 10.3389/fonc.2022.946580 (PMC9402900; doi:10.3389/fonc.2022.946580)
Supplement: Supplementary file 1 [file DataSheet_1.docx]

**Development and validation of a deep learning model for breast lesion segmentation and characterization in multiparametric MRI**

Jingjin Zhu1,6†, Jiahui Geng2†, Wei Shan3†, Boya Zhang1,6, Huaqing Shen2, Xiaohan Dong4, Mei Liu5, Xiru Li6*, Liuquan Cheng4*

**MRI Protocol**

Both internal and external MRI examinations were running the same protocol: 3.0 Tesla MRI scanner with eight-channel phase array breast coil (Signa HDxt, Discovery 750 or 750/w, General Electric Healthcare, Milwaukee, USA). The imaging protocol, lasting for 18 minutes, included 4 pulse sequences: DWI, T2 weighted imaging (T2WI), T1 weighted imaging (T1WI), and DCE. All sequences were spatially matched in axial view, and field of view 320mm320mm and 190mm in Z-axis coverage. The b-value of DWI was 0 and 800 or 1000sec/mm2 in three orthogonal diffusion gradients, IR 250ms for fat suppression, TR 5400ms, minimum TE, matrix 128×128. The T2WI used IDEAL for fat suppression, TR 5000ms, TE 68ms, matrix 320256. Both T1WI and DCE used the same VIBRANT (Volume Imaging for BReast AssessmeNT), a spectral-selective inversion recovery (SPECIAL) and 3-dimensional spoiled gradient recall sequence. SPECIAL option was disabled for non-fat-suppression T1WI. The T1WI and DCE had the exact same geometric location: an isotropic spatial resolution of 1.0mm×1.0mm×1.0mm, 192 partitions in axial view, minimum TR/TE, flip angle 120. The DCE scan repeated 6 continuous phases without interruption, each of which lasted 120 seconds. After completing the pre-contrast phase, the contrast agent (0.5M Gd-DTPA) was injected through an antecubital vein at a rate of 2ml/sec, 0.1mmol/kg body weight, and with 20ml saline flushing.

**Supplementary Table 1. The results of the McNemar test for different diagnostic methods**

| Method | | DCE&DWI | | P value |
| --- | --- | --- | --- | --- |
|  |  | B | M |  |
| DWI | B | 112 | 45 | 0.057 |
|  | M | 66 | 200 |  |
| DCE | B | 141 | 27 | 0.260 |
|  | M | 37 | 218 |  |
| BI-RADS by Radiologists  23&45 | B | 64 | 3 | ＜0.001 |
|  | M | 114 | 242 |  |
| BI-RADS by Radiologists  234&5 | B | 128 | 24 | 0.003 |
|  | M | 50 | 221 |  |

DWI, Diffusion-weighted imaging; DCE, dynamic contrast enhancement; B, benign; M, malignant
